# DHEA and Its Metabolites Reduce the Cytokines Involved in the Inflammatory Response and Fibrosis in Primary Biliary Cholangitis

**DOI:** 10.3390/ijms24065301

**Published:** 2023-03-10

**Authors:** Małgorzata Blatkiewicz, Katarzyna Sielatycka, Katarzyna Piotrowska, Ewa Kilańczyk

**Affiliations:** 1 Department of Histology and Embryology, Poznan University of Medical Sciences, 61-781 Poznań, Poland; 2 Faculty of Exact and Natural Sciences, Institute of Biology, University of Szczecin, 70-415 Szczecin, Poland; 3 Sanprobi Sp. z.o.o. Sp.k., 70-535 Szczecin, Poland; 4 Department of Physiology, Pomeranian Medical University in Szczecin, 70-111 Szczecin, Poland; 5 Department of Medical Biology, Pomeranian Medical University in Szczecin, 70-111 Szczecin, Poland

**Keywords:** DHEA, interleukins, estrogens, steroidogenesis, PBC

## Abstract

Dehydroepiandrosterone (DHEA) is an abundant steroid and precursor of sex hormones. During aging, the reduction in DHEA synthesis causes a significant depletion of estrogens and androgens in different organs, such as the ovaries, brain, and liver. Primary Biliary Cholangitis (PBC) is a cholestatic liver disease that begins with immune-mediated bile duct damage, and is followed by liver fibrosis, and finally, cirrhosis. PBC primarily affects postmenopausal women, with an average age of diagnosis of 65 years, but younger women are also affected. Here, we analyzed the levels of DHEA, estradiol (E2), and estriol (E3) in the PBC sera of females at an age of diagnosis under 40 (*n* = 37) and above 65 (*n* = 29). Our results indicate that in PBC patients at an age of diagnosis under 40, E2 was significantly lower compared to that in healthy women. In contrast, the levels of DHEA and E3 were in a normal range. Furthermore, ELISA assays revealed that in PBC patients at an age of diagnosis above 65, the levels of DHEA, E2, and E3 significantly declined in comparison to those in younger patients. In addition, flow cytometry analysis showed that the level of IL-8 significantly decreased while the level of TNF-α increased in older PBC patients compared to younger ones. Moreover, we showed for the first time that the sulfonated form of DHEA, DHEA-S, reduces the levels of both pro-inflammatory interleukins, IL-8 and TNF-α, in PBC-like cholangiocytes (H69-miR506), while it diminishes the level of the pro-fibrotic interleukin, IL-13, in hepatocytes (Hep-G2). Finally, we demonstrated that the expression of the pro-fibrotic agent TGF-β significantly increased in both the early (F0–F3) and cirrhotic (F4) stages of PBC, and this elevation was accompanied by higher α-SMA expression.

## 1. Introduction

Primary Biliary Cholangitis predominantly affects middle-aged females, with a typical clinical outcome occurring during the postmenopausal period [1,2]. Much less attention in research has been paid to young women with PBC. Although there are potentially significant differences in the clinical expression of PBC between older and younger patients, the basic approach to therapy is the same in both groups. Here, we want to focus on young PBC patients (at an age of diagnosis below 40) to see how DHEA and estrogen levels affect the development of PBC.

A breakdown of the biliary immune tolerance of the E2 subunit of the pyruvate dehydrogenase complex and the faultily activated immune response seems to play a central role in PBC pathogenesis. The primary targets of damage in this disease are cholangiocytes, which are epithelial cells lining the biliary tree. Under normal conditions, cholangiocytes remain in a quiescent state, while in cholestasis, they can be activated and secrete various cytokines and chemokines, e.g., IL-6, IL-8, and TNF-α [3,4,5]. IL-8 is a pro-inflammatory factor and it is released by neutrophils and macrophages in response to various stimuli, including steroids, inflammatory signals, and environmental stresses [6]. Specific cytokines, such as TNF-α and IL-1β, are potent inducers of IL-8 expression in a variety of cells, such as macrophages and epithelial cells [7,8].

In the terminal stage of liver fibrosis, cirrhosis is a common result of chronic diseases regardless of etiology [9]. The primary evidence of fibrosis is the activation of the main source of the extracellular matrix (ECM), hepatic stellate cells (HSCs), which finally lead to ECM overgrowth. The most crucial cytokine that activates HSCs is transforming growth factor beta (TGF-β). However, in patients with chronic liver diseases, the effect of TGF-β in liver fibrogenesis seems to be etiology-dependent [10]. In addition to TGF-β, interleukin 13 (IL-13) is recognized as a critical pro-fibrotic cytokine in many organs, including the liver [11].

The idea that estrogens can influence the clinical course of PBC came from several studies showing that estrogens can modulate the humoral and cellular immune response by stimulation of Th2 anti-inflammatory cytokines (IL-10, IL-4, and TGF-β) and inhibition of Th1 pro-inflammatory cytokines (IL-12, TNF-α, and interferon γ (IFN-γ)) [12,13,14,15]. Recent findings suggest that estrogens may influence the course of PBC by directly modulating the proliferation or apoptosis [5] and pathological changes of cholangiocytes [16,17,18]. On the other hand, the levels of these estrogens depend on the amount of their precursor, i.e., DHEA. Over their lifetime, the peak of the DHEA level in females appears at 15–19 years of age, and then its level decreases markedly with age [19,20]. DHEA is mainly synthesized in the adrenal zona reticularis and can be rapidly converted to endpoint sex hormones, i.e., androgens and estrogens. These reactions occur in the ovaries, brain, and liver and can be catalyzed by tissues in specific enzymatic systems [19]. DHEA also exists as a sulfated ester known as DHEA-S. The latter is mainly present in the circulation due to its binding affinity for albumin [21]. In addition, steroid sulfotransferase 2A1 (SULT2A1) can increase circulating DHEA-S by sulfating DHEA. It was reported earlier that the level of SULT2A1 significantly decreases in liver tissues of PSC patients (another type of cholestatic liver disease) [22]. Moreover, the serum levels of DHEA-S significantly decline in PBC and PSC patients and are usually correlated with fatigue [23,24] and low quality of life [25].

Since DHEA plays an indispensable role in maintaining steroid hormone balance in the body, we measured the levels of DHEA, E2, and E3 in two groups of PBC patients. The first group included young women diagnosed under 40 who were still menstruating. In contrast, the second group involved women diagnosed over 65 who were in the postmenopausal period. Our interest was mainly focused on females in whom the diagnosis was made pretty early on, i.e., before 40 years of age, to evaluate whether the DHEA level could influence PBC development. We also checked the correlation between DHEA, E2, E3, and cytokines (IL-1β, IL-8, IL-10, IL-12p70, IL-13, and TNF-α) in the PBC sera of the examined patients.

As estrogens are known for their anti-inflammatory activities [26,27,28], they may be beneficial in inhibiting the progression of chronic inflammatory liver diseases. The effects of the estrogen precursor DHEA and its metabolites (DHEA-S, E2, DHT, adione, and adiol) on IL-8, IL-13, and TNF-α levels were evaluated in: (i) normal cholangiocytes (NHC and H69); (ii) PBC-like cholangiocytes with the overexpression of miR-506 (H69-miR506); and (iii) hepatocytes (Hep-G2 cells). In addition, the expression of two proteins involved in the fibrogenesis process (TGF-β and α-SMA) was examined in cirrhotic PBC liver tissues.

## 2. Results

### 2.1. The Levels of DHEA and Estrogens (E2 and E3) in Sera of PBC Patients at Different Ages of Diagnosis

Since DHEA is a crucial precursor of steroid hormones such as estradiol (E2) and estriol (E3), and its level declines with age, we checked the DHEA, E2, and E3 levels in two groups of female PBC patients at different ages of diagnosis (under 40 and over 65).

**Figure 1:**
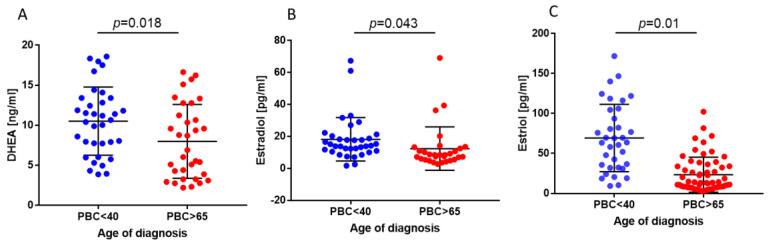
Reduced levels of DHEA, E2, and E3 in sera of female PBC patients at an age of diagnosis over 65. The levels of DHEA (**A**), E2 (**B**), and E3 (**C**) were measured using appropriate ELISA assays in sera of females under 40 (*n* = 37) and over 65 (*n* = 29) at PBC diagnosis.

First, we found that in younger females at an age of PBC diagnosis under 40, the level of E2 (18.1 ± 13.3 pg/mL) was below the ranges of E2 (>70 pg/mL in each menstruation phase) for healthy women at the same age [29], whereas the levels of DHEA (10.8 ± 4.3 ng/mL) and E3 (69.3 ± 41.5 pg/mL) were still in the normal range for a healthy person, i.e., DHEA: 2–9 ng/mL and E3: <120 pg/mL (Figure 1A–C). Furthermore, the ELISA assay revealed that the levels of DHEA and both E2 and E3 were significantly lower (20% reduction, *p* = 0.018; 25% reduction, *p* = 0.043; and 45% reduction, *p* = 0.01) in older female patients at an age of PBC diagnosis over 65 in comparison to younger patients (Figure 1A–C).

### 2.2. The Levels of Pro-, and Anti-Inflammatory Interleukins and TNF-α in Sera of PBC Patients

**Figure 2:**
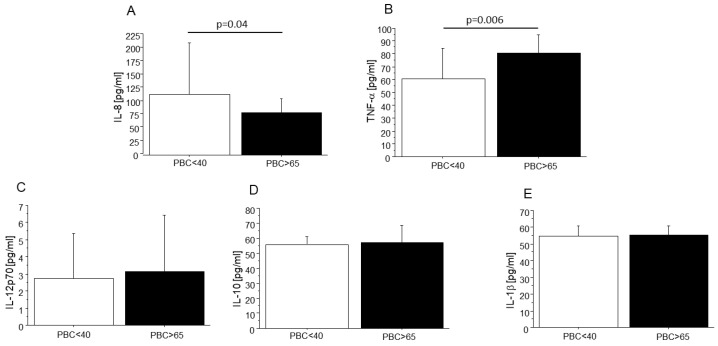
The levels of cytokines (IL-1β, IL-8, IL-12p70, IL-10,and TNF-α) in sera of PBC patients. Sera of PBC patients at an age of diagnosis under 40 (*n* = 37) and over 65 (*n* = 29) were incubated with the appropriate antibody conjugated with a fluorophore, and then flow cytometry analysis (cytometric bead assay (CBA)) (**A**–**E**) was performed. Levels of all cytokines are expressed in pg/mL. Results are presented as mean ± SD.

Since inflammatory response is an important common mechanism contributing to PBC development, the levels of anti- (IL-10) and pro-inflammatory interleukins (IL-1β, IL-8, and IL-12p70) and TNF-α were evaluated and compared in sera of females at an age of diagnosis under 40 and over 65. Flow cytometry analysis showed that in the serum of PBC patients at an age of diagnosis over 65, the level of IL-8 was significantly lower (40% reduction, *p* = 0.04; Figure 2A) while the level of TNF-α was significantly higher (20% elevation, *p* = 0.006; Figure 2B) in comparison to these levels in PBC patients at an age of diagnosis under 40. Then, the correlation coefficient parameter (*r*) was analyzed. In both groups of PBC patients (younger and older), strong positive correlations between IL-1β and both TNF-α and IL-10, as well as between IL-10 and TNF-α, were observed. Furthermore, weak, positive correlations between IL-12p70 and both IL-1β and TNF-α were presented only in young PBC patients (see Table 1).

In the case of the older group of PBC patients, estriol (E3) was negatively correlated with IL-10 (*r* = −0.423, *p* = 0.027), IL-1β (*r* = −0.523, *p* = 0.0026), and TNF-α (*r* = −0.401, *p* = 0.027) (see Table 1). Moreover, estradiol (E2) was negatively correlated only with IL-8 (*r* = −0.542, *p* = 0.002). Finally, IL-8 was positively correlated with both IL-1β (*r* = 0.382, *p* = 0.036) and IL-10 (*r* = 0.397, *p* = 0.039) (see Table 1).

### 2.3. The Effect of DHEA and Its Metabolites on IL-8 and TNF-α Levels in Cholangiocytes and Hepatocytes

**Figure 3:**
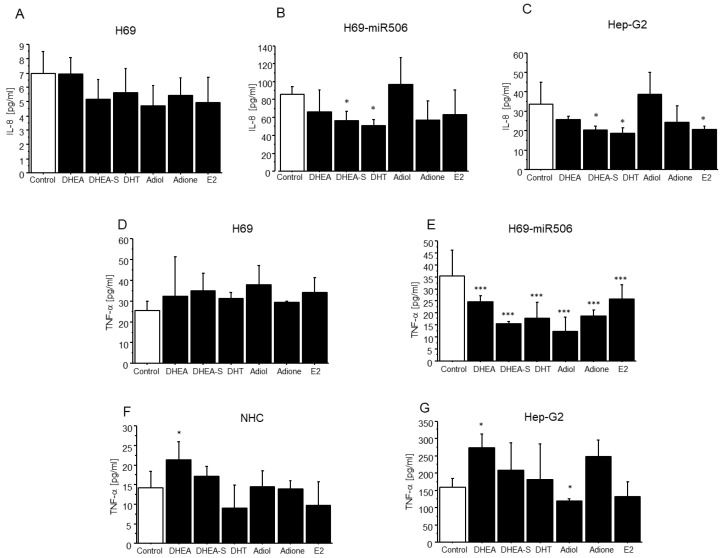
DHEA and its metabolites affect the IL-8 (**A**–**C**) and TNF-α (**D**–**G**) levels in cholangiocytes and hepatocytes. Cells were incubated with DHEA, DHEA-S, DHT, adiol, adione, and E2. After 24 h, the levels of IL-8 and TNF-α were measured using appropriate ELISA assays. Results are presented as mean ± SD (*n* = 3); * *p* < 0.05, *** *p* < 0.001.

As estrogens are known for their anti-inflammatory activities, we tested whether the estrogen precursor DHEA and its metabolites affect IL-8 and TNF-α levels in control cholangiocytes (H69, NHC), PBC-like cholangiocytes (H69-miR506), and hepatocytes (Hep-G2). For this purpose, cells were incubated with 1 nM of DHEA, DHEA-S, E2, and adiol, or 1 μg/μL of adione and DHT. After 24 h, both DHEA-S and DHT diminished the level of IL-8 in both PBC-like cholangiocytes (H69-miR506) and hepatocytes (Hep-G2) (*p* < 0.05; Figure 3B,C). Moreover, E2 reduced the level of IL-8 only in hepatocytes (Hep-G2) (*p* < 0.05; Figure 3C). In the case of TNF-α, it was significantly diminished by DHEA and its metabolites were only reduced in PBC-like cholangiocytes (H69-miR506) (*p* < 0.001; Figure 3E). In addition, one of DHEA’s metabolites, adiol, significantly reduced the level of TNF-α in human hepatocytes (*p* < 0.05; Figure 3G). Finally, DHEA enhanced the level of TNF-α in NHC and Hep-G2 cells (*p* < 0.05; Figure 3F,G).

### 2.4. The Effect of DHEA and Its Metabolites on the Level of Pro-Fibrotic IL-13

**Figure 4:**
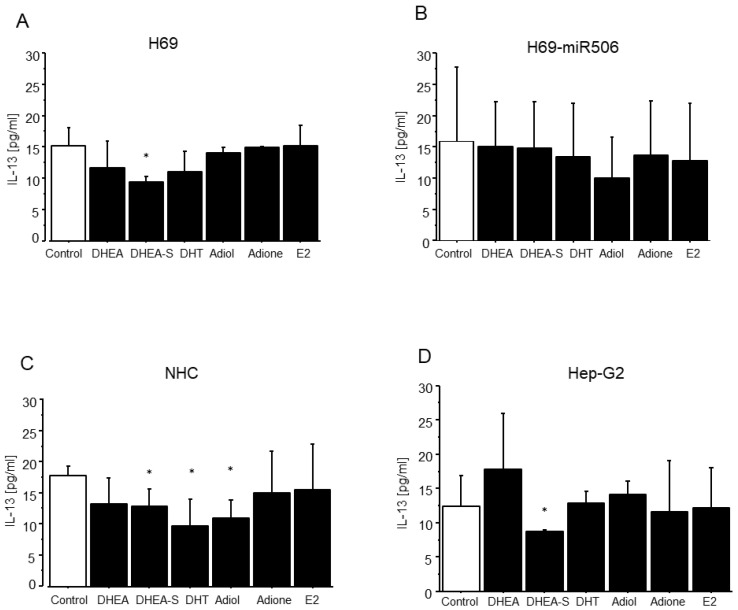
DHEA-S, DHT, and adiol affect the interleukin 13 level in normal human cholangiocytes (H69 and NHC) and hepatocytes (Hep-G2). Cells (**A**–**D**) were incubated with DHEA, DHEA-S, DHT, adiol, adione, and E2. After 24 h, the level of IL-13 was measured using an appropriate ELISA assay, * *p* < 0.05, *n* = 3.

Since IL-13 was correlated with enhanced fibrosis, we checked how DHEA and its metabolites influence the level of IL-13 in cholangiocytes and hepatocytes. We showed for the first time that DHEA-S significantly diminished the level of IL-13 in normal human cholangiocytes (H69 and NHC) (*p* < 0.05; Figure 4A,C) and hepatocytes (Hep-G2) (*p* < 0.05; Figure 4D). Moreover, DHT and adiol significantly reduced the level of IL-13 in normal human cholangiocytes (NHC) (*p* < 0.05; Figure 4B). Neither DHEA nor its metabolites changed IL-13 levels in PBC-like human cholangiocytes (H69-miR506) (Figure 4D).In addition, our unpublished ELISA data revealed that the level of IL-13 was not significantly changed in sera of older female patients at an age of PBC diagnosis over 65 in comparison to younger patients. However, there was a significant negative correlation between IL-13 and both IL-1β (*r* = −0.507, *p* = 0.003) and TNF-α (*r* = −0.390, *p* = 0.032) and positive correlation between IL-13 and E3 (*r* = 0.572, *p* = 0.0007) in PBC patients at an age of diagnosis above 65 (see Table 1).

### 2.5. Expression of TGF-β and α-SMA in Control Non-Cirrhotic and Cirrhotic Human Liver Tissues (PBC and PSC)

**Figure 5:**
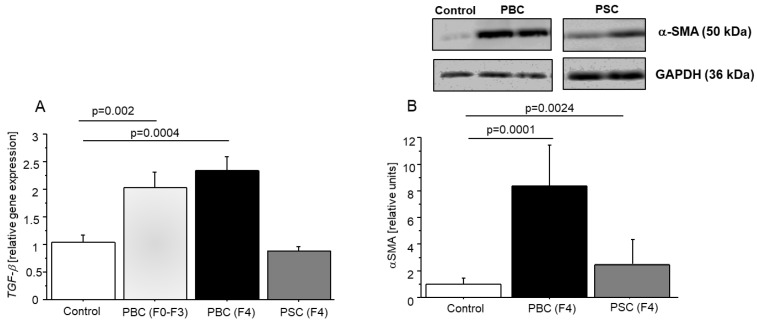
The elevated expression of TGF-β and the level of α-SMA in cirrhotic (PBC, PSC) and control liver tissues. The relative gene expression of TGF-β in liver tissues at early stage (F0–F3; *n* = 18) and cirrhotic stage of PBC (F4; *n* = 10) and PSC (F4; *n* = 10), which was measured by quantitative PCR (real-time PCR) (**A**). Western blot analysis revealed the protein expression level of α-SMA in cirrhotic PBC (F4; *n* = 10) and PSC (F4; *n* = 10) tissues (**B**).

Since a close link between TGF-β and liver fibrosis was demonstrated earlier, we investigated the expression of TGF-β at the mRNA level at an early stage (F0–F3) of PBC development. Moreover, the expression of TGF-β in cirrhotic liver tissues of PBC (characterized by the highest fibrotic score, i.e., F4) was also determined. The expression of TGF-β was significantly elevated in PBC in both the early and advanced (cirrhotic) stages of the disease. The analysis showed that at the early stage of PBC (F0–F3), the expression of TGF-β indicates a 2-fold increase, with a similar enhancement at the advanced stage of PBC (F4) (Figure 5A). To confirm that elevated expression of TGF-β concerned only PBC we used cirrhotic liver tissues (fibrotic score of F4) from patients suffering from PSC (Primary Sclerosis Cholangitis), which is another type of cholestatic liver disease. The expression of TGF-β in PSC cirrhotic tissues was comparable to that in the controls.

Then, we investigated the expression of the α-SMA protein (the main marker of fibrosis) in control and cirrhotic liver tissues (PBC and PSC) using an immunoblot. The α-SMA expression was 8.6-fold higher in cirrhotic PBC patients and 3-fold higher in PSC patients compared to the control (*p* = 0.0001 and *p* = 0.0024, respectively; Figure 5B).

## 3. Discussion

**Figure 6:**
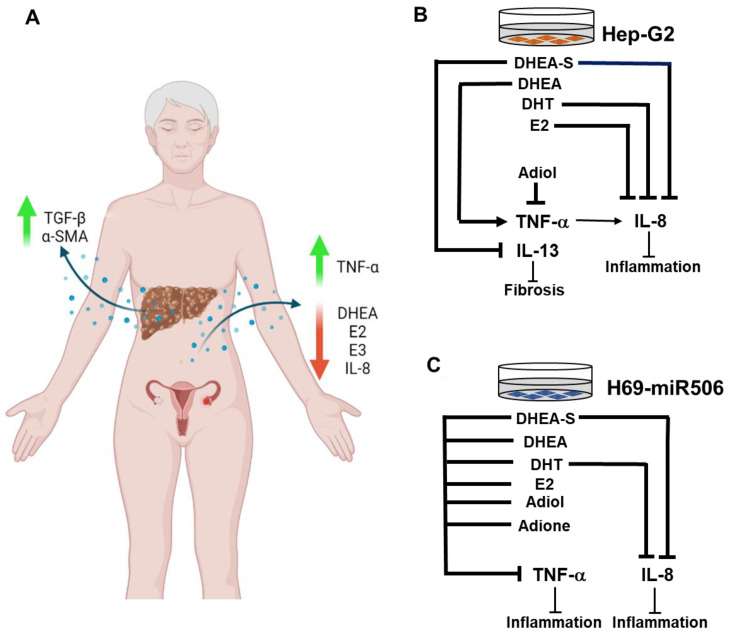
The potential involvement of DHEA and its metabolites in modulation of inflammation and fibrosis. The level of steroid hormones (DHEA, E2, and E3) and cytokines (IL-8 and TNF-α) in the serum of PBC patients at an age of diagnosis above 65 (**A**). The expression of TGF-β and α-SMA in cirrhotic PBC livers (**A**). The effect of DHEA and its metabolites (DHEA-S, E2, and DHT) on inflammation and fibrosis in hepatocytes (Hep-G2) (**B**) and PBC-like cholangiocytes with overexpression of miR-506 (H69-miR506) (**C**), (see the discussion section for more details).

The role of estrogens in autoimmune diseases has been extensively studied. Since DHEA facilitates its effects through the conversion to endpoint sex hormones, in the present study, the levels of DHEA, estradiol (E2), and estriol (E3) were evaluated in PBC sera of patients at an age of diagnosis under 40 and over 65. Our results indicated that E2 levels in young PBC patients at an age of diagnosis under 40 were significantly lower compared to those in healthy women [29]. In contrast, the levels of DHEA and E3 were in a normal range. We also found that in PBC patients at an age of diagnosis over 65, the levels of DHEA, E2, E3, and IL-8 significantly decreased. In contrast, the level of TNF-α significantly increased compared to that in younger PBC patients (females at an age of diagnosis under 40). Moreover, we showed for the first time that the sulfonated form of DHEA, DHEA-S, can reduce IL-8 and TNF-α levels in PBC-like cholangiocytes (H69-miR506) and the IL-13 level in hepatocytes (Hep-G2). Finally, we demonstrated that the expression of TGF-β was significantly increased in both early (F0–F3) and cirrhotic (F4) stages of PBC (see Figure 6).

In PBC patients under 40, the level of estrogen (E2) was below the reference range for fertile women. As reported earlier, low levels of estradiol [30] or testosterone [31,32] could predict mortality in chronic liver diseases. In addition, we found that the level of DHEA in both younger and older PBC patients was still within the reference range for healthy females. This could be due to there being a significantly higher cholesterol level (substrate for DHEA synthesis) in PBC patients (see the characteristics of PBC patients, Table 2). On the other hand, bioactive DHEA may be recovered locally from DHEA-S through conversion by steroid sulfatase (STS), and its expression is significantly elevated in PBC cirrhotic liver tissues [5].

Additionally, the lower level of DHEA in older PBC patients at an age of diagnosis above 65 could be caused by aging and an elevated level of TNF-α, as reported previously [33]. Mauduit et al. demonstrated that TNF-α inhibits DHEA synthesis mainly by decreasing the availability of cholesterol, which is the initial substrate required for steroidogenesis. Besides the lower level of DHEA in older patients at an age of diagnosis under 65, we observed significantly lower levels of both E2 and E3, which were negatively correlated with pro-inflammatory interleukins (IL-8, TNF-α, and IL-β) (see Table 1). This may suggest that the presence of estrogens can improve the PBC outcome. Of note, in young PBC patients at an age of diagnosis under 40, there was a strong positive correlation only between pro-inflammatory interleukins, suggesting that immune response, not estrogen level, is the first evidence of the development of PBC in young women, which is in line with the observation that young PBC patients suffer from other autoimmune diseases. Our results show that the inflammation process is continuous throughout the duration of PBC.

It was postulated that the pathological starting point of PBC is the autoimmune-mediated injury of the small bile duct. To figure out whether interleukins play a role in the development of PBC, the levels of pro- (IL-1β, IL-8, IL-12p70, and TNF-α) and anti-inflammatory (IL-10) interleukins were evaluated in both PBC patients at an age of diagnosis under 40 and above 65. Our results demonstrated that the level of TNF-α was increased in the sera of PBC patients at an age of diagnosis above 65, as was reported previously [34]. Generally, the elevated level of TNF-α reflects the severity of PBC [35,36,37]. Furthermore, our results demonstrated that the IL-8 level was diminished in the sera of PBC patients at an age of diagnosis above 65 and was positively correlated with IL-10. This finding is in line with previous research showing that IL-10 and TGF-β downregulate IL-8 production in response to IL-1β or TNF-α [38]. Moreover, IL-8’s role in PBC development is well documented [34,39]. It was reported earlier that a higher level of IL-8 may reflect liver cirrhosis progression [40] and correlates with the development of PBC [41,42].

To the best of our knowledge, we showed for the first time that DHEA and its metabolites significantly decreased TNF-α levels, mainly in H69-miR506 cholangiocytes. It was reported earlier that these cells possess the PBC phenotype and are characterized by an increased level of TNF-α [43]. Numerous studies have illustrated the anti-inflammatory action of DHEA and DHEA-S [44,45,46,47]. DHEA exerts its anti-inflammatory effects primarily by modulating either the effect or production of pro-inflammatory cytokines. It was reported earlier that DHEA can act as a potent modulator of TNF-α expression in the liver and cecum [48]. Furthermore, our results demonstrated that in PBC-like (H69-miR506) cholangiocytes, DHEA-S and E2 significantly reduced the level of IL-8, another pro-inflammatory cytokine involved in the progression of PBC. In addition, DHEA and its metabolites did not affect the level of IL-8 and TNF-α in control cholangiocytes (H69), probably due to its unstimulated stage.

Our results confirmed the occurrence of fibrosis in both PBC and PSC since immunoblot analysis revealed a higher level of α-SMA protein (the primary marker of fibrosis initiation) in cirrhotic liver (PBC and PSC) tissues. Additionally, we confirmed the involvement of TGF-β in fibrogenesis processes, particularly in PBC, as the expression of TGF-β was significantly increased at both the early (F0–F3) and cirrhotic (F4) stages of PBC development. Moreover, even in cirrhotic tissue, we observed the permanent expression of fibrogenic factors, which exacerbates the PBC disease. Besides TGF-β, IL-13 is a critical pro-fibrotic cytokine in different organs, including the liver [11]. However, in contrast to the lung [49], IL-13 induced fibrosis independently of TGF-β in the liver [50]. Since IL-13 possesses a strong pro-fibrotic nature, we checked whether DHEA and its metabolites could modulate the level of IL-13. Indeed, our results demonstrated that DHEA-S significantly reduced the level of IL-13 in both hepatocytes (Hep-G2) and cholangiocytes (NHC and H69), whereas DHT and adiol reduced the IL-13 level in cholangiocytes (NHC). Our results are in line with recently reported findings that DHEA has strong anti-fibrotic effects in normal lung fibroblasts and in an ex vivo model of human precision-cut lung slices (PCLS) [51]. Furthermore, DHEA prevents the incidence of NASH in female mice by reducing hepatic steatosis, fibrosis, and inflammation [52].

The main limitation of this paper is that we did not know in which phase of the menstrual cycle the young women were during serum sample collection. We realize that concentrations of circulating estrogens widely vary throughout life, and the specificity and sensitivity of estrogen immunoassays are not perfect. However, we possess a unique collection of PBC patients’ sera, which enables us to figure out the mechanism of PBC development in young women (26–39 years old). Our results demonstrate that the disease course depends mainly on the inflammatory response in younger PBC patients. In contrast, in older PBC patients, besides the immune response, the levels of estrogens play a role in PBC progression.

## 4. Materials and Methods

### 4.1. Serum Samples and Cell Culture

Sixty-six female PBC patients were divided into two groups based on an age of diagnosis under 40 and above 65 years old. The clinical and biochemical parameters of the examined patients are shown in Table 2. Human cholangiocytes (NHC, H69, and H69-miR506) were cultured as previously described [43]. Human hepatocytes (Hep-G2) were purchased from American Type Culture Collection (Manassas, VA, USA). Hep-G2 cells were grown in Eagle’s Minimum Essential Medium containing 10% fetal bovine serum (FBS; Gibco, Waltham, MA, USA), 100 U/mL of penicillin, and 100 µg/mL of streptomycin (Sigma-Aldrich, St. Louis, MO, USA). Cultures were maintained in the presence of 5% CO_2_ at 37 °C. Cells were incubated with 1 nM of DHEA, DHEA-S, E2, and adiol, or 1 μg/μL of adione and DHT. After 24 h, appropriate ELISA assays were performed.

### 4.2. Tissue Sample Preparation

Molecular analysis was carried out in human liver tissue samples from different groups of patients according to disease and the degree of liver fibrosis. Liver tissue specimens were collected by undergoing percutaneous liver biopsies from non-cirrhotic PBC patients (F0–F3; *n* = 18). Liver tissue specimens were collected from explanted livers of patients with histologically proven cirrhosis with PBC (F4; *n* = 10) and PSC (F4; *n* = 10). Control liver tissues (*n* = 12) were obtained from large margin liver resections of colorectal metastases, and no microscopic changes in liver disease were identified by a pathologist. Percutaneous needle liver biopsies (non-cirrhotic PBC) were stored in RNAlater (Applied Biosystems, Carlsbad, CA, USA). Liver tissue specimens were collected as tissue blocks (~1 cm^3^), which were immediately frozen in liquid nitrogen and stored at −75 °C until use. For analysis, samples were powdered and homogenized in an appropriate lysis buffer to extract either total RNA or protein.

### 4.3. ELISA Assays

The concentrations of DHEA (Elabscience, #EL-0115), E2 (Cayman, #501890), E3 (Elabscience, #EL-0156), and IL-13 (Elabscience, #EL-H0104) in sera of patients with PBC were measured with ELISA kits according to the manufacturer’s instructions. Serum samples for the measurement of E2 were extracted from a methanol mixture, which was followed by evaporation to dryness under an inert gas. Then, samples were reconstituted with an assay buffer and measured immediately. In addition, IL-13 (Elabscience, #EL-H0104), TNF-α (Elabscience, #EL-H0109), and IL-8 (Elabscience, #EL-H6008) in culture cells were tested with corresponding ELISA kits. Briefly, after discarding the medium and washing the samples three times in PBS, the cells were incubated with pre-chilled PBS, and the freeze–thaw process was repeated to obtain cell lysates.

### 4.4. Flow Cytometry

The BD Cytometric Bead Array (CBA) Human Inflammatory Cytokine Kit was used to quantitatively measure interleukin-8 (IL-8), interleukin-1β (IL-1β), interleukin-10 (IL-10), tumor necrosis factor-alpha (TNF-α), and interleukin-12p70 (IL-12p70) protein levels in sera of PBC patients (see the characteristics of the patients in Table 2). All analyses were performed according to the manufacturer’s procedure (BD Cytometric Bead Array Human Inflammatory Cytokine Kit, #551811). Briefly, serum samples (25 μL) were diluted and incubated with beads conjugated with appropriate antibodies against the examined interleukin. Then, a detection reagent was added, which provided a fluorescent signal in proportion to the amount of the bound analyte. Thirty minutes later, the complexes (capture bead, analyte, and detection reagent) were measured using a CytoFLEX LX (Beckman Coulter, Brea, CA, USA) flow cytometer, and data were analyzed using CytExpert 2.0 data analysis software (Beckman Coulter, Brea, CA, USA).

### 4.5. Quantitative Real-Time PCR (qRT-PCR) Analysis

Total RNA was extracted from tissue samples (50 mg) using the RNeasy Mini Kit (Qiagen, Hilden, Germany). Isolated RNA quantity and quality were determined using an Epoch spectrophotometer (BioTek, Winooski, VT, USA). Subsequently, cDNA was prepared from 20 ng of total RNA at a 20 μL reaction volume using the SuperScriptTM First-Strand Synthesis System for RT-PCR (#11904018, Invitrogen, Carlbad, CA, USA). Quantitative real-time PCR was performed using the 7500 Fast Real-Time PCR System (Applied Biosystems, ThermoFisher Scientific, Waltham, MA, USA) with the following pre-validated TaqMan gene expression assays: TGFβ1 (Hs00998133_m1) and 18S (Hs99999901_s1). Each 20 μL reaction mix included the TaqMan Gene Expression Master Mix (Applied Biosystems, ThermoFisher Scientific, Waltham, MA, USA) and 2 μL of cDNA. Each sample was analyzed simultaneously in two replicates. Calculations were performed using the ΔΔCt relative quantification method. The Ct values for each sample were normalized to the mean value obtained for the endogenous control gene of 18S ribosomal RNA.

### 4.6. Immunoblot Analysis

Frozen liver tissues were lysed in a RIPA buffer supplemented with the cOmplete™ Mini Protease Inhibitor Cocktail (Roche, Basel, Switzerland) and phosphatase inhibitors (PhosSTOP EASYpack; Roche, Basel, Switzerland). Extracted proteins (60 μg) were resolved in SDS polyacrylamide gels and then transferred onto a PVDF membrane using the semi-dry transfer (Immobilon-P; Millipore). The primary antibody against α-smooth muscle actin (#19245, Cell Signaling, 3 Trask Lane, Danvers, MA, USA; 1:1000 dilution) was used. After washing with TBST buffer, the secondary antibody ECL Anti-Rabbit IgG HRP (NA9340, GE Healthcare, Piscataway, NJ, USA) was used. Protein loading was normalized using GAPDH (sc-25778 + HRP; Santa Cruz, Inc., Portland, Oregon, USA; 1:5000 dilution). The bands were visualized using a chemiluminescent detection reagent with a long-lasting signal (Amersham, GE Health Life Sciences, Piscataway, NJ, USA). Image and densitometry analyses were performed using MicroChemi Imaging Systems and GelQuant software (DNR Bio-Imaging, Modi’in-Maccabim-Re’ut, Israel).

### 4.7. Statistical Analysis

All the presented data were analyzed using the StatView software version 5 (SAS Institute Inc., Carry, NC, USA). Statistical differences were determined by Fisher’s PLSD test and presented as a bar plot, indicating mean ± SD. The z-test was used for correlation analysis. A value of *p* < 0.05 was considered statistically significant.

## 5. Conclusions

The pathogenesis of PBC remains incompletely understood, and effective treatment is lacking. This paper provides an important insight into the essentials of the effect of DHEA and its metabolites on the levels of IL-8, IL-13, and TNF-α in normal and PBC-like (H69-miR506) cholangiocytes. We believe that further studies of DHEA–DHEA-S involvement in PBC pathogenesis and development will help to gain important and highly clinically relevant insights.

## Figures and Tables

**Table 1:** Significant correlation between cytokines and estrogens in sera of PBC patients at an age of diagnosis under 40 (*n* = 37) and above 65 (*n* = 29).

Parameters	Patients	
	PBC < 40 Correlation Coefficient (*r*)	PBC > 65 Correlation Coefficient *(r)*
IL-1β and TNF-α	0.767 (*p* < 0.0001)	0.934 (*p* < 0.0001)
IL-1β and IL-10	0.549 (*p* = 0.001)	0.729 (*p <* 0.0001)
IL-1β and IL-12p70	0.328 (*p* = 0.046)	
IL-10 and TNF- α	0.737 (*p <* 0.0001)	0.486 (*p* = 0.009)
TNF-α and IL-12p70	0.414 (*p* = 0.008)	
E3 and IL-1β		−0.523 (*p* = 0.0026)
E3 and IL-10		−0.423 (*p* = 0.027)
E3 and TNF-α E3 and IL-13		−0.401 (*p* = 0.027) 0.572 (*p* = 0.0007)
E2 and IL-8		−0.542 (*p* = 0.002)
IL-13 and IL-1β		−0.507 (*p* = 0.0037)
IL-13 and TNF-α		−0.390 (*p* = 0.0325)
IL-8 and IL-1β		0.382 (*p* = 0.036)
IL-8 and IL-10		0.397 (*p* = 0.039)

**Table 2:** Baseline clinical characteristics of analyzed PBC patients (mean ± SD).

Parameters	Patients	
	PBC < 40 (*n* = 37)	PBC > 65 (*n* = 29)
Age of diagnosis (years, range)	34 ± 4 (24–39)	74 ± 5 (67–82)
Age of the study (years, range)	39 ± 7 (29–56)	76 ± 6 (68–87)
ALP (IU/L: normal: 40–120)	384 ± 335	320 ± 198
Bilirubin (mg/dL: normal: 0.2–1.0)	1.7 ± 2.4	2.6 ± 3.9
Total cholesterol (mg/dL: normal: 114–200)	237 ± 86	229 ± 45
Liver fibrosis stages (F0–F3/F4)	32/5	20/9

## Data Availability

The data presented in this study are available upon reasonable request.

## Abbreviations

DHEA—dehydroepiandrosterone; ECM—extracellular matrix; E2—17β-estradiol; E3—estriol; DHT—5-dihydrotestosterone; HSCs—hepatic stellate cells; NASH—nonalcoholic steatohepatitis; PAGE—polyacrylamide gel electrophoresis; PBS—phosphate-buffered saline; PBC—Primary Biliary Cholangitis; PSC—Primary Sclerosis Cholangitis; TGF-β—transforming growth factor beta; α-SMA—alpha-smooth muscle actin.
